# Acute Respiratory Failure and Active Bleeding Are the Important Fatality Predictive Factors for Severe Dengue Viral Infection

**DOI:** 10.1371/journal.pone.0114499

**Published:** 2014-12-02

**Authors:** Kamolwish Laoprasopwattana, Wanwipa Chaimongkol, Pornpimol Pruekprasert, Alan Geater

**Affiliations:** 1 Department of Pediatrics, Faculty of Medicine, Prince of Songkla University, Hat Yai, Songkhla, Thailand; 2 Epidemiology Unit, Faculty of Medicine, Prince of Songkla University, Hat Yai, Songkhla, Thailand; Duke-National University of Singapore Graduate Medical School, Singapore

## Abstract

**Objective:**

To determine the outcome of severe dengue viral infection (DVI) and the main dengue fatality risk factors.

**Study design:**

The medical records of patients aged <15 years admitted to Songklanagarind Hospital in southern Thailand during 1989–2011 were reviewed. Patients who had dengue hemorrhagic fever (DHF) grades III–IV, organ failure (cardiovascular, respiratory, liver, renal or hematologic), impaired consciousness, or aspartate aminotransferase more than 1,000 units/L, were classified as having severe DVI. To determine the fatality risk factors of severe DVI, the classification trees were constructed based on manual recursive partitioning.

**Results:**

Of the 238 children with severe DVI, 30 (12.6%) died. Compared to the non-fatal DVI cases, the fatal cases had higher rates of DHF grade IV (96.7% vs 24.5%), repeated shock (93.3% vs 27.9%), acute respiratory failure (ARF) (100% vs 6.7%), acute liver failure (ALF) (96.6% vs 6.3%), acute kidney injury (AKI) (79.3% vs 4.5%), and active bleeding requiring blood transfusion (93.3% vs 5.4%), all p<0.01. The combined risk factors of ARF and active bleeding considered together predicted fatal outcome with sensitivity, specificity, and negative and positive predictive values of 0.93 (0.78–0.99), 0.97 (0.93–0.99), 0.99 (0.97–1.00), and 0.82 (0.65–0.93), respectively. The likelihood ratios for a fatal outcome in the patients who had and did not have this risk combination were 32.4 (14.6–71.7) and 0.07 (0.02–0.26), respectively.

**Conclusion:**

Severe DVI patients who have ARF and active bleeding are at a high risk of death, while patients without these things together should survive.

## Introduction

More than 50 million cases of dengue fever (DF) and several hundred thousand cases of dengue hemorrhagic fever (DHF), with an overall fatality rate of approximately 0.2–2%, occur each year in tropical countries [1].

Previous case control and/or retrospective studies have found that patients with dengue shock syndrome (DSS) with multi-organ failure involving acute liver failure (ALF), acute respiratory failure (ARF), acute kidney injury (AKI) and/or hematologic failure (active bleeding) are at risk of lethal DHF/DSS [2]–[9]. In addition, studies in Thai children have found that obese children had a higher risk of having severe DVI than normal weight children [5], [10], [11].

There has been to date no published study on a system to predict a higher risk of fatality in children with severe DVI, thus this study was undertaken to determine fatality predictive factors in children with severe DVI.

## Methods

We retrospectively reviewed the medical records of all children (<15 years of age) diagnosed with severe DVI admitted from January 1989–December 2011 in Songklanagarind Hospital, the major tertiary care center for the 14 provinces of southern Thailand. Permission from the institutional review board of Prince of Songkla University was obtained prior to conducting the study. Our study involved the use of patient medical data, from which any information that could specifically identify any patient was removed before the analysis was performed, and thus the institutional review board waived the need for written informed consent from the participants.

DVI was diagnosed according to the criteria of the World Health Organization (WHO) [1]. Primary or secondary DVI was diagnosed if there was a four-fold increase of hemagglutination inhibition test and the titers were ≤1∶1,280 and ≥1: 1∶2,560, respectively. DHF was diagnosed if the patient fulfilled all of the WHO criteria: acute febrile illness; hemorrhagic manifestation; thrombocytopenia (<100,000 platelets/mm^3^); and evidence of plasma leakage as determined by hemoconcentration (hematocrit increased above baseline by ≥20%), pleural or abdominal effusion (as revealed by radiography or another imaging method), or hypoalbuminemia. DHF grade I was diagnosed if the patient met all of the DHF criteria without evidence of circulatory failure. DHF grade II was diagnosed if the patient had evidence of a bleeding disorder. DHF grades III or IV (DSS) were diagnosed if the patient met all of the DHF criteria and there was also evidence of impending (narrow pulse pressure, <20 mmHg) or profound circulatory failure.

Demographic characteristics and known potential risk factors for disease severity were recorded, including age, sex, underlying diseases, weight standard deviation score (WSDS), obesity (WSDS>2), and severity of DVI according to the WHO criteria [1]. Respiratory failure was defined by severe hypoxemia requiring a mechanical ventilator. Hematologic failure was defined by active bleeding requiring packed red cells and/or other blood components to control. AKI was defined by a sudden increase in serum creatinine (Cr) levels >2 mg/dL or a serum Cr concentration >2 times previous or subsequent values and that was also higher than the upper limit of normal values for the patient's age [12]. ALF was defined by the rapid development of severe acute liver injury with impaired synthetic function (international normalized ratio (INR) ≥1.5) and encephalopathy in a patient with no history of liver disease. To determine the severity of plasma leakage, coagulopathy, hepatitis, and renal impairment, the highest and lowest levels of hematocrit (Hct), highest white blood cell count (WBC), the lowest platelet count, the highest prothrombin (PT) and activated partial thromboplastin times (aPTT), highest levels of aspartate aminotransferase (AST), alanine aminotransferase (ALT), total bilirubin (TB), direct bilirubin (DB), blood urea nitrogen (BUN), and Cr, and the lowest levels of albumin and serum bicarbonate were obtained from the medical records. Patients who had DSS, organ failure or impaired consciousness (including seizure) or AST >1,000 unit/dL were classified as severe DVI [1].

## Statistical analysis

Data were evaluated using descriptive statistics (mean and standard deviation, median and interquartile range (IQR), or frequency and percentage, as appropriate). Comparisons between severe DVI patients who died and survived were made using Student's *t*-test or Mann-Whitney *U*-test for normally distributed and non-normally distributed continuous variables, respectively. Chi-square test or Fisher's exact test were used for comparisons of categorical data. To determine the fatality risk factor of severe DVI, the classification trees were constructed based on manual recursive partitioning guided by consideration of the practical application. Stata version 10 was used for statistical analysis.

## Results

### Characteristics and clinical course of severe DVI

During the 22-year period, 3,630 patients aged <15 years were diagnosed with DVI needing hospitalization; of these, severe DVI was diagnosed in 238 (6.6%) patients, and 30 (0.8%) of these patients subsequently died.

Of the 238 patients who had severe DVI, 73 (30.7%) had been referred from another hospital, 127 (53.4%) were male and the mean age was 8.5±3.7 years (range 3 months to 15.0 years). DSS was diagnosed in 228 patients (95.8%), and the 10 non-DSS patients (DF or DHF grades I or II) were diagnosed as severe DVI because of organ failure in 6 patients, seizure in 3 patients and AST >1,000 unit/L in 1 patient. Of the 6 patients with organ failure, 3 had multi-organ failure (2 and 1 patients had 2 and 3 organ failures, respectively). None of the non-DSS patients died.

Of the 113 patients who had convalescent plasma samples confirming DVI, primary and secondary DVI were diagnosed in 8 (7.1%) and 105 (92.9%) patients, respectively. All 7 patients who were younger than 2 years of age had primary DVI. Of the 30 fatal DVI cases, 16 had convalescent plasma sample which confirmed the diagnosis, and all except one, aged 6 months, had secondary DVI.

### Clinical course and outcomes of DSS

Of the 228 patients with DSS, DHF grades III and IV were diagnosed in 148 and 80 patients, respectively. The mean duration from the first day of fever until shock developed in these patients was 4.7±1.4 days (range 3–13 days). Organ failure was found in 67 (29.4%) patients and 48 (21.0%) of these had multi-organ failure.

Of the 148 patients with DHF grade III, organ failure was found in 20 (13.5%) and 10 (6.8%) had multi-organ failure which involved 2, 3, and 4 organs in 5, 4, and 1 patients, respectively. Respiratory failure, ALF, AKI, and active bleeding were found in 8 (5.4%), 6 (4.0%), 10 (6.8%), and 8 (5.4%) patients, respectively. One of 148 patients died from a nosocomial infection (*Pseudomonas stutzeri* septicemia) involving multi-organ failure.

Of the 80 patients with DHF grade IV, organ failure was found in 42 (52.5%) patients and 37 (46.2%) patients had multi-organ failure which involved 2, 3, and 4 organs in 8, 8, and 21 patients, respectively, and 29 (37.2%) of these patients died from multi-organ failure. ARF, ALF, AKI, and active bleeding were found in 35/80 (43.8%), 33/79 (41.8%), 26/79 (32.9%), and 33/80 (41.3%) patients, respectively.

### Risk factors of fatality in severe DVI

Of the 30 patients who died, the median time (IQR) from shock to death was 5.0 (2.0–8.3) days; 6 (25.0%) patients died within 24 hours of first going into shock. All of these 30 had respiratory failure and all had multi-organ failure.

To determine the fatality risk factors in severe DVI, we compared the clinical characteristics and possible fatality risk factors between the severe DVI patients who died and those who survived. Fatal DVI patients had significantly higher WSDS, a higher proportion of DHF grade IV, repeated shock, and abnormal laboratory findings including lower levels of lowest hematocrit and serum bicarbonate and higher levels of highest Hct, WBCs, DB, TB, AST, ALT, ALP, albumin, BUN and Cr, and PT and aPTT (Table 1). The proportion of patients with organ failure, including ARF requiring mechanical ventilation, ALF, AKI, or hematologic failure (active bleeding requiring blood transfusion) were significantly higher in the DVI patients who died (Table 2).

**Table 1:** Comparing characteristics and laboratory results of patients with severe DVI between those who died and those who survived.

Characteristic	Died (N = 30)	Survived (N = 208)	P*
Male, n (%)	16 (53.3)	111 (53.4)	0.997
Age, years, mean ± SD	8.0±3.5	8.5±3.7	0.432
Referred, n (%)	23 (76.7)	50 (24.0)	<0.001
Underlying disease, n (%)	5 (16.7)	34 (16.6)	>0.999
WSDS, median (IQR)	0.67 (0.07–1.95), n = 29	0.00 (−1.20–1.15)	0.009
Nutritional status	n = 29		0.334
Obese, n (%)	7 (24.1)	29 (13.9)	
Underweight, n (%)	1 (3.4)	12 (5.8)	
Normal bodyweight, n (%)	21 (72.4)	167 (80.3)	
Diarrhea, n (%)	13 (56.5), n = 23	65 (33.5), n = 194	0.029
Final diagnosis			<0.001
Dengue fever, n (%)	0	1 (0.5)	
DHF gr. I, n (%)	0	4 (1.9)	
DHF gr. II, n (%)	0	5 (2.4)	
DHF gr. III, n (%)	1 (3.3)	147 (70.7)	
DHF gr. IV, n (%)	29 (96.7)	51 (24.5)	
Shock >1 times	28 (93.3)	58 (27.9)	<0.001
Laboratory results			
Lowest hematocrit, %, mean ± SD	27.2±7.2, n = 28	32.8±6.4, n = 205	<0.001
Highest hematocrit, %, mean ± SD	47.3±7.2, n = 29	44.6±5.4	0.013
Highest WBC/mm^3^, median (IQR)	18,015 (10,407–24,725), n = 28	6,600 (4,600–10,300), n = 175	<0.001
Lowest platelet/mm^3^, median (IQR)	17,000 (12,500–30,000), n = 29	29,500 (18,250–46,500)	0.007
Highest DB, mg/dL, median (IQR)	3.3 (1.2–6.7), n = 27	0.2 (0.1–0.5), n = 137	<0.001
Highest TB, mg/dL, median (IQR)	5.1 (2.1–9.4), n = 27	0.4 (0.3–0.9), n = 138	<0.001
Highest AST, units/L, median (IQR)	9,945 (2,752–14,180), n = 29	270 (119–837), n = 144	<0.001
Highest ALT, units/L, median (IQR)	1,874 (1,400–2,943), n = 29	124 (88–416), n = 143	<0.001
Lowest albumin, gram/dL, mean ± SD	2.5±0.6, n = 28	2.9±0.7, n = 133	0.029
Highest ALP, units/L, median (IQR)	165 (101–223), n = 28	124 (88–166), n = 137	0.019
Highest BUN, mg/dL, median (IQR)	37.9 (22.6–65.0), n = 29	14.7 (11.2–21.0), n = 180	<0.001
Highest Cr, mg/dL, median (IQR)	3.5 (1.2–5.3), n = 29	0.7 (0.5–0.8), n = 182	<0.001
Lowest HCO_3_, mEq/L, mean ± SD	13.1±4.7, n = 29	18.3±4.0, n = 182	<0.001
Highest PT, seconds, median (IQR)	30.1 (23.9–43.8), n = 29	13.5 (11.4–15.9), n = 120	<0.001
Highest aPTT, seconds, median (IQR)	81.6 (58.6–100), n = 29	41.7 (36.0–55.5), n = 119	<0.001

*Fisher's exact test, *t*-test, or Mann-Whitney *U*-test, as appropriate.

WSDS: weight standard deviation score; DHF: dengue hemorrhagic fever; PT: prothrombin time aPTT: activated partial thromboplastin time; DB: direct bilirubin; TB: total bilirubin.

ALT: serum alanine aminotransferase; AST: serum aspartate aminotransferase; ALP: Alkaline phosephatase; Cr: serum creatinine; BUN: blood urea nitrogen.

**Table 2:** Organ failure in severe DVI - a comparison between patients who died and patients who survived.

	Died (N = 30)	Survived (N = 208)	
Organ failure	n, (%)	n, (%)	P
Respiratory failure	30 (100)	14 (6.7)	<0.001
Acute liver failure	28 (96.6), n = 29	13 (6.3)	<0.001
Active bleeding	28 (93.3)	16 (7.7)	<0.001
Acute kidney injury	23 (79.3), n = 29	16 (7.7)	<0.001
Number of organ failures	N = 29		<0.001
Single organ failure	0	22 (10.6)	
Multiorgan failure (2 organs)	4 (13.8)	13 (6.3)	
Multiorgan failure (3 organs)	7 (24.1)	8 (3.8)	
Multiorgan failure (4 organs)	18 (62.1)	0	

The mortality rates of the severe DVI patients who were hospitalized during the first 11 years of the study period (Jan 1989–Dec 1999) and the last 11years (Jan 2001–Dec 2011) were not significantly different (8/53 (15.1%) vs 22/185 (11.9%), p = 0.54, respectively). The proportions of patients who had an underlying disease were not different between the surviving and non-surviving patients. Of the 30 patients with fatal DVI, 2 patients had an underlying disease that made them more vulnerable to a fatal outcome, one with angiofibromatosis causing massive nosebleed from the original tumor and the other with glucose-6-phosphate dehydrogenase deficiency causing intravascular hemolysis.

Using recursive partitioning, we found that the combination of ARF and active bleeding occurring together was the major risk factor of a fatal outcome with high sensitivity, specificity, negative predictive value (NPV), and positive predictive value (PPV) (Table 3). The likelihood ratios (LR) for fatal outcome of severe DVI patients who had and did not have both ARF and active bleeding together were 32.4 (14.6–71.7) and 0.07 (0.02–0.26), respectively.

**Table 3:** Sensitivity, specificity, NPV and PPV of combined ARF and active bleeding in prediction of fatal DVI.

ARF and active bleeding together	Died	Survived	Predictive value (95% CI)
Yes	28	6	PPV = 0.82 (0.65–0.93)
No	2	202	NPV = 0.99 (0.97–1.00)
Sensitivity = 0.93 (0.78–0.99)		Specificity = 0.97 (0.93–0.99)	

NPV, negative predictive value; PPV, positive predictive value; ARF; acute respiratory failure.

### The outcome of severe DVI patients who survived

Of the 208 patients who survived, 43 (20.7%) had organ failure (35/43 (81.4%) had 1 or 2 organ failures, no patient had failure of 4 organs). Patients who had organ failure were hospitalized longer than those who had no organ failure (mean ± SD of 11.7±8.9 days vs 4.5±2.6 days, p<0.001).

The average duration (median, IQR) of intubation in the 14 patients who had respiratory failure was 5.5 (2.5–7.5) days, the average duration of unconsciousness in the 13 patients with ALF was 5 (2–16) days, the average duration of bleeding in the 16 patients who had active bleeding was 1 (1–3) day, and the average time before the Cr levels returned to normal in the 16 patients with AKI was 11 (3–22) days. All of the patients with severe DVI who survived were discharged without consequent chronic organ failure.

## Discussion

The overall fatality rate of severe DVI in our patients was 12.6%; patients who had neither ARF nor active bleeding survived.

We found that children who had both ARF and active bleeding had a high chance to develop fatal DVI. A previous study found that the respiratory section of the Sequential Organ Failure Assessment (SOFA) test had a high accuracy in predicting fatal outcomes in severe DVI [13]. We found, as in this study, that ARF was the major factors which could predict fatal DVI. However, the SOFA test is generally used to evaluate critically ill adults rather than children, so has some limitations with younger children. In addition, the SOFA coagulation analysis uses platelet counts to determine the severity of coagulation failure and bilirubin levels to determine the severity of hepatic failure, but a recent study found that these particular two tests cannot reliably identify high risk of fatal DVI in adult patients with severe DVI [13]. In our study, most patients with severe DVI had a low platelet count and most patients with ALF had a high AST level rather than a high TB level, and most patients had TB lower than 10.0 mg/dL. Our findings support the findings of a study by Juneja et al., which concluded that platelet counts and TB levels are not useful in predicting fatal DVI [13].

Other tools have been developed to attempt to predict mortality outcomes in sick children, notably the Pediatric Risk of Mortality (PRISM) and the Pediatric Index of Mortality (PIM) tests [14], [15]. The key mortality predictive factors of both PRISM and PIM are shock, ARF, and unconsciousness as determined by a coma score and pupillary response to a bright light. We did not include a coma score because levels of consciousness are difficult to evaluate in young children and cannot be evaluated in sedated patients. Also, fixed pupil dilatation can be detected in nearly dead patients in some cases.

The major causes of death in our study were DHF grade IV and multi-organ failure, while other studies have found the causes of fatal DVI in adult patients were not only DSS and subsequent multi-organ failure but also concurrent or secondary bacteremia and underlying diseases contributing to death [6]–[8]. We found only one patient of the 30 who died had a secondary bacterial infection that might have been contributory, and two others had an underlying disease that might have contributed to an unfavorable outcome.

In general, DVI fatality rates vary according to various factors, such as the age group or severity of DVI, the availability of intensive medical care, and the experience of the medical team. The overall fatality rate of DVI varies from 0.2–2% in tropical countries [1]. For example, Kalayanarooj et al. found that 8/4,532 (0.2%) of hospitalized Thai children with DVI died [11], and although we found a higher mortality rate of 30/3,630 (0.8%) in our hospitalized DVI children, we also note that our institution is the major referral center in southern Thailand, and the mortality rate of non-referred patients was 7/3,557 (0.2%) patients, similar to the Kalayanarooj et al. study.

Preventing DVI patients from continuing to DSS, and preventing those who do develop DSS from developing organ failure, are the keys to minimizing fatal DVI. A previous study found that after the implementation of a dengue guideline, the mortality rate decreased from 7.4% to 3.1% in hospitalized children in a referral hospital [16]. A prospective cohort study in Vietnamese children found a low mortality rate of 8/1,719 (0.5%) in DSS cases; in this study, all patients were treated at a pediatric intensive care unit (PICU) with prompt management from an experienced team [17]. In our study, all DSS patients except those who died at the emergency room (ER) were also treated at our PICU, with an overall DSS mortality rate of 30/228 (13.2%). However, if calculating only non-referred DSS patients, and DHF grade III patients, the mortality rates would be 7/159 (4.4%) and 1/148 (0.7%) patients, respectively. Of the 7 non-referred DSS patients, 2 cases were already in profound shock when they arrived at the ER.

We found that fatal DVI patients had higher WBCs than those who survived, which was similar to previous studies [6], [8]. The higher WBCs in fatal DVI patients could be explained by the high levels of inflammatory cytokines and stress hormones, and concurrent bacterial infection [18], [19]. Although documented bacteremia cases in a previous study [8] and our study were low, empirical treatment with antibiotics is common in treating DVI with multi-organ failure, especially in cases with respiratory failure or ALF, because these patients are more vulnerable to a nosocomial infection. The higher inflammatory cytokines may also cause diarrhea, which we found in a higher proportion of our fatal DVI patients [20].

Anders et al. found that Vietnamese girls had a higher risk of DSS and death than boys [21]. Previous studies in adult patients have found that males were more likely to have severe DVI (65% vs 35%) [13], and males also had a higher proportion of fatal DVI (19/28, 67.9%) than females [9]. However, another study found a higher proportion of fatal DVI in females (9/10, 90.0%) [22]. Our study found no gender bias in mortality rates.

The study had two limitations. First, it was a retrospective study, thus there could have been some missing information, and the precise times when organ failure developed might be unreliable in some cases. In addition, only 113/238 (47.5%) had a convalescent plasma sample to confirm acute DVI. Although 52.5% of the patients had only a clinical diagnosis without serology-confirmed DHF, the high specificity in our patients of the WHO criteria for DHF diagnosis (95–99%) can be seen as highly supportive of our conclusion that most of the patients in our study had DHF [23]–[25]. Secondly, some laboratory investigations such as LFT or a coagulogram to determine ALF or BUN and Cr to determine AKI were not performed in one patient who died at the emergency room, or in severe DVI patients who had no risk factors for, or clinical profiles suggesting, organ failure. To overcome these limitations and to validate whether the combined risk of ARF and active bleeding can be used to predict fatal outcomes, a multi-center prospective study would need to be performed.

In conclusion, severe DVI patients who had both ARF and active bleeding in our retrospective analysis had high sensitivity, specificity, NPV, and PPV in identifying children at high-risk of dying, thus we believe that children with this combination of risk factors should be provided with extra attention to reduce the mortality rate. Severe-DVI children with neither of these risk factors should survive. To overcome the limitations of this study, a prospective multi-center study is needed.

## Supporting Information

Data S1 **Severe dengue viral infection.**
(XLSX)
